# Estimating Extrinsic Dyes for Fluorometric Online Monitoring of Antibody Aggregation in CHO Fed-Batch Cultivations

**DOI:** 10.3390/bioengineering4030065

**Published:** 2017-07-24

**Authors:** Karen Schwab, Friedemann Hesse

**Affiliations:** Institute of Applied Biotechnology, Biberach University of Applied Science, 77781 Biberach, Germany; hesse@hochschule-bc.de

**Keywords:** antibody aggregation, ANS, bioprocess monitoring, Bis-ANS, fluorescence spectroscopy, CHO

## Abstract

Multi-wavelength fluorescence spectroscopy was evaluated in this work as tool for real-time monitoring of antibody aggregation in CHO fed-batch cultivations via partial least square (PLS) modeling. Therefore, we used the extrinsic fluorescence dyes 1-anilinonaphthalene-8-sulfonate (ANS), 4,4′-bis-1-anilinonaphthalene-8-sulfonate (Bis-ANS), or Thioflavin T (ThT) as medium additives. This is a new application area, since these dyes are commonly used for aggregate detection during formulation development. We determined the half maximum inhibitory concentrations of ANS (203 ± 11 µmol·L^−1^), Bis-ANS (5 ± 0.5 µmol·L^−1^), and ThT (3 ± 0.2 µmol·L^−1^), and selected suitable concentrations for this application. The results showed that the emission signals of non-covalent dye antibody aggregate interaction superimposed the fluorescence signals originating from feed medium and cell culture. The fluorescence datasets were subsequently used to build PLS models, and the dye-related elevated fluorescence signals dominated the model calibration. The soft sensors based on ANS and Bis-ANS signals showed high predictability with a low error of prediction (1.7 and 2.3 mg·mL^−1^ aggregates). In general, the combination of extrinsic dye and used concentration influenced the predictability. Furthermore, the ThT soft sensor indicated that the intrinsic fluorescence of the culture might be sufficient to predict antibody aggregation online.

## 1. Introduction

One objective of process analytical technology (PAT) is to improve process understanding at any process step by identifying the related critical process parameters (Food and Drug Administration 2004). One possibility to gain a better understanding of cultivation processes in bioreactors is the implementation of online monitoring based on spectroscopic soft sensors [1]. However, spectroscopic data cannot be interpreted by univariate statistical techniques, which are mostly applied to access industrial data sets [2]. Multivariate data analysis has to be used to extract the required information from the huge dataset [3,4]. The most common approach is to use partial least square regression (PLSR) to interpret the data. The underlying algorithm is capable of extracting a linear dependency of an x-dataset and the corresponding y-dataset [5]. Offline measured response variables such as substrate, metabolites, and viable cell concentration can be used as y-dataset. The x-dataset mostly consists of large, noisy, and highly redundant data recorded by the spectroscopic sensors. The resulting PLSR model can be used for online and real-time prediction of the respective response variables. Among the available spectroscopic techniques [6,7,8], 2D fluorescence spectroscopy (2DFS) proved to be a reliable method which allowed online monitoring of viable cell concentrations as well as product and metabolite concentrations in mammalian cell batch and fed-batch cultivations with non-fluorescent feed [9]. Ohadi et al. (2014) described that it is possible to predict key process parameters at-line in Chinese hamster ovary cell (CHO) shake flask cultures [10]. Schwab et al. (2016) proved that it was possible to predict fluorescent and non-fluorescent key process parameters online in CHO fed-batch cultivations with highly fluorescent feed medium. So far, however, none of the existing studies have focused on online monitoring of product quality parameters during upstream processing (USP). One example of an important quality parameter which is reported to be an issue throughout the whole monoclonal antibody (mAb) production process is product aggregation [11]. MAb aggregates are known to reduce drug performance and can cause anaphylactic reactions [12]. It was shown by various groups that mAb aggregation can already occur during USP, since aggregation can be influenced by medium components, ionic strength, pH control, and temperature shift [13,14,15]. Paul et al. (2015) analyzed mAb aggregates directly in CHO cell culture supernatant via size exclusion chromatography, and reported roughly 77% aggregated mAb [16]. A high aggregate content during USP might eventually reduce the overall yield and increase the burden on downstream processing. The intrinsic fluorescence of proteins is based on the aromatic amino acids tryptophan, tyrosine, and phenylalanine, and Ohadi et al. (2015) proved that it is possible to use the intrinsic fluorescence of the mAb in combination with chemometric modeling in order to identify aggregate levels in purified protein samples at-situ [17,18]. Therefore, fluorometric online monitoring of mAb aggregation in CHO fed-batch cultivations should be possible in principle. However, it was suspected that the fluorescence signals of medium, feed medium, and cells could possibly outbalance the emission signal changes related to mAb aggregate formation. Therefore, the fluorescence dyes 4,4′-bis-1-anilinonaphthalene-8-sulfonate (Bis-ANS) and its monomeric analogue 1-anilinonaphthalene-8-sulfonate (ANS) along with thioflavin T (ThT) were used in this study. These extrinsic dyes show low fluorescence signals in aqueous solution, but the fluorescence signals increase upon non-covalent binding to partially unfolded or aggregated proteins [16,19]. Extrinsic dyes are usually applied as fluorescent markers in tissue sections, protein purification, and formulation development [20,21,22,23]. However, Paul et al. (2015) have already applied ANS and Bis-ANS for the detection of aggregated mAb in cell-free CHO culture supernatant. They reported that specific regions of the recorded excitation emission matrices (EEM) showed increased fluorescence signals which were directly related to increased aggregate concentrations [16]. Furthermore, these dyes can be solved in water instead of ethanol or DMSO like Congo red, Nile red, and DCVJ [23]. Nevertheless, none of these dyes have been used thus far as additive to mammalian cell cultivations in order to measure protein aggregation.

In this context, the current study aims to demonstrate that 2DFS in combination with extrinsic fluorescence dyes can be used for real-time monitoring of mAb aggregation during CHO fed-batch cultivations. Therefore, half maximum inhibitory concentrations (IC50) had to be determined for the extrinsic fluorescence dyes ANS, Bis-ANS, and ThT in CHO cultivations. The dyes created additional emission signals in the EEM which were used to build PLSR models for the prediction of mAb aggregate concentrations. The challenge was to estimate the concentrations for each dye that allowed the calibration of a reliable PLSR model. The resulting soft sensors were compared regarding their predictive capability and susceptibility to disturbance by feed additions. Based on the results, we evaluated which extrinsic dye was most suitable and can be used for real-time monitoring of mAb aggregation in CHO fed-batch cultivations.

## 2. Materials and Methods 

### 2.1. Cell Line, Medium, and Culture Conditions

The CHO DG44 cell line expressing an aggregation-prone mAb was used as model system. The cells were seeded at 2–4 × 10^5^ mL^−1^ in SFM4CHO medium (GE Healthcare, Chicago, IL, USA), incubated at 37 °C and 5% CO_2_, and passaged every 3–4 days. Glucose and l-glutamine (Life Technologies, Carlsbad, CA, USA) were added to the medium upon usage. All chemicals were ordered from Roth (Karlsruhe, Germany) if not stated otherwise.

### 2.2. IC50 Experiments

Stock solutions of 3 mmol·L^−1^ ANS, 1 mmol·L^−1^ Bis-ANS, and 2 mmol·L^−1^ ThT (Sigma-Aldrich, Taufkirchen, Germany) in SFM4CHO were prepared and sterile filtered with 0.2 µm (Phenomenex, Torrance, CA, USA). The following dye concentration ranges were used for toxicity evaluation: (**a**) 0–400 µmol·L^−1^ ANS; (**b**) 0–80 µmol·L^−1^ Bis-ANS; and (**c**) 0–20 µmol·L^−1^ ThT. Exponentially growing CHO cells were spun down, diluted in fresh medium, and seeded with a final cell concentration of 4 × 10^5^ mL^−1^ (*n* = 3) in 24-well plates with a final volume of 2 mL (Greiner Bio-one, Frickenhausen, Germany). The fluorescence dyes were directly supplied to the wells together with the medium containing 5 g·L^−1^ glucose and 4 mmol·L^−1^ l-glutamine. The plates were covered with breath seals (Greiner Bio-one, Frickenhausen, Germany) in addition to the lit and incubated in a shaker with a maximum deflection of 4 mm (Kuhner, Basel, Switzerland). The final viable cell concentrations and viabilities were determined after 72 h cultivation using a FACSCalibur flow cytometer (DB Bioscience, San Jose, CA, USA), following the propidium iodide staining protocol proposed by Cummings and Schnellmann [24].

### 2.3. Fed-Batch Cultures with Fluorescence Dyes

Three fed-batch cultivations were performed for each dye, and the details are listed in Table 1. The fed-batch cultivations were inoculated with a cell concentration of 10 × 10^5^ mL^−1^. A 2-L benchtop bioreactor BIOSTAT^®^ Bplus (Sartorius, Göttingen, Germany) was used, and the temperature was kept constant at 37 °C. The dissolved oxygen saturation was kept at 60% through sparging with an aeration rate of 0.25 vvm, while pH 7 was maintained through CO_2_ or 1 M NaOH addition. The culture was stirred at 100 rpm, and the glucose target concentration for feeding was set between 0.8–1.2 g·L^−1^. Cell Boost 6 (4% *w*/*v*) (GE Healthcare, Chicago, IL, USA) was solved in deionized water and supplemented to the bioreactor continuously as glucose feed. The feed rate was adjusted depending on the glucose consumption rates. Likewise, l-glutamine was diluted in SFM4CHO (100 mmol·L^−1^) and added as a bolus feed when required to keep the concentrations ≥0.8 mmol·L^−1^. The extrinsic dyes were added to the bioreactor 67 ± 4 h after the inoculation, with a final concentration of either 100 µmol·L^−1^ ANS, 2 µmol·L^−1^ Bis-ANS, or 2 µmol·L^−1^ ThT. Glucose and l-glutamine feeds contained the same fluorescence dye concentration as the cell culture. 

### 2.4. Offline Analytics

The antibody concentration was determined with protein A HPLC using a POROS^®^ 20 µm column (Thermo Fisher Scientific, Waltham, MA, USA) and the UltiMate 3000 system (Thermo Fisher Scientific, Waltham, MA, USA). The mAb aggregate concentration in the cell culture samples was determined with SE-HPLC using the Agilent 1100 system (Agilent Technologies, Santa Clara, CA, USA). A MAbPac SEC-1 column (Thermo Fisher Scientific, Waltham, MA, USA) was used, following the method described by Paul et al. (2014) for the direct determination of mAb aggregate concentration in cell culture samples [25]. l-glutamine and glucose concentrations were determined enzymatically with the KonelabTM 20 XT (Thermo Fisher Scientific, Waltham, MA, USA) using the l-glutamine kit (Thermo Fisher Scientific, Waltham, MA, USA) and the Glucose HK kit (Thermo Fisher Scientific, Waltham, MA, USA), respectively. Cells were counted using a Cedex XS analyzer (Roche, Basel, Switzerland) and trypan blue exclusion.

### 2.5. Online Data Collection

The multi-wavelength EEMs were recorded with the BioView^®^ sensor (Delta, Hørsholm, Denmark), using a fiber optic assembly for the 19 mm port. A gain of 1300 and a measurement interval of 15 min were set, and the glass vessels were covered with blackout material. Excitation wavelengths from 270–550 nm and emission wavelengths ranging from 310–590 nm in 20 nm steps were used to record the EEMs. The resulting fluorescence datasets were exported vectorized into two-way arrays containing 120 excitation/emission wavelength pairs (λ_ex/em_) per EEM. A detailed description of the instrument can be found elsewhere [26,27,28].

### 2.6. Chemometric Modeling and Data Preprocessing

All chemometric methods were performed using MatLab version 8.4.0 (MathWorks, Natick, MA, USA) in combination with the PLS-toolbox version 7.9.5 (Eigenvector Research Ing., Manson, WA, USA) and a detailed description of the chemometric method can be found elsewhere [5,29]. The EEMs of all fed-batch cultivations were preprocessed with multiplicative signal correction (MSC). This was followed by an external parameter orthogonalization (EPO) filtering method with one principal component [30] (Table 2) for cultivations containing ANS and Bis-ANS. No further preprocessing was applied to datasets from cultivations containing ThT. The SIMPLS algorithm was used for PLSR modeling. EEMs and corresponding offline measured aggregate concentrations were used as input for the model generation. Only EEMs and corresponding offline data recorded after dye addition were used for PLSR modeling. The samples were randomly split into a calibration and a validation dataset (66–34%) using the onion method. The resulting models were selected with the aim of minimizing the RSME of calibration (RMSEC), cross-validation (RMSECV), and prediction (RMSEP) with the preferably lowest possible number of latent variables (LV) [29]. MAb aggregate concentrations were predicted based on all EEMs recorded during the cultivation and the selected chemometric models. Quality parameters of the selected models are listed in Table 2.

## 3. Results

### 3.1. IC50 Experiments

**Figure 1:**
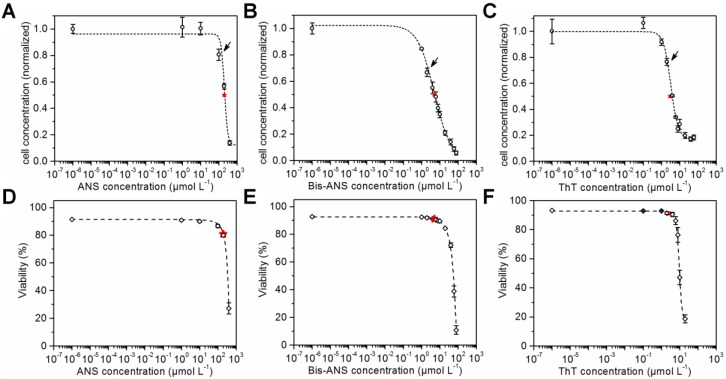
Cell concentration after 72 h cultivation in the presence of ANS (IC50 of 203 ± 11 µmol·L^−1^), Bis-ANS (IC50 of 5 ± 0.5 µmol·L^−1^) and ThT (IC50 of 3 ± 0.2 µmol·L^−1^) (**A**–**C**) and the corresponding viabilities (**D**–**F**) are shown. A sigmoid curve fit was performed in order to determine the corresponding IC50s (indicated by red stars). Arrows indicate the dye concentrations that were used later in the fed-batch processes.

CHO cells were seeded in microtiter plates and cultivated in the presence of ANS, Bis-ANS, and ThT. Viable cell concentrations and viabilities were determined after a cultivation time of 72 h using flow cytometry and propidium iodide staining. The viable cell concentrations were plotted against the logarithmic concentration of the particular dye. Sigmoid curves were fitted to the data points, and the respective IC50s were calculated (Figure 1A–C). ANS with a calculated IC50 of 203 ± 11 µmol·L^−1^ was less toxic than the other dyes. In comparison, an IC50 of 5 ± 0.5 µmol·L^−1^ was calculated for its dimeric analogue Bis-ANS, and an even lower IC50 of 3 ± 0.2 µmol·L^−1^ was calculated for ThT. The sigmoid curve fit was also used for the viability data (Figure 1D–F). This made the comparison of cell concentrations and cell viabilities at half maximum inhibitory dye concentration possible. A viability of 80% was calculated for the IC50 concentration of ANS. Furthermore, a viability of 91% was determined for an IC50 concentration of Bis-ANS or ThT. This showed that the cell growth was already reduced compared to the control experiment without dye, but the viability was not affected in the same way.

### 3.2. Online Monitoring Based on Extrinsic Fluorescence

**Figure 2:**
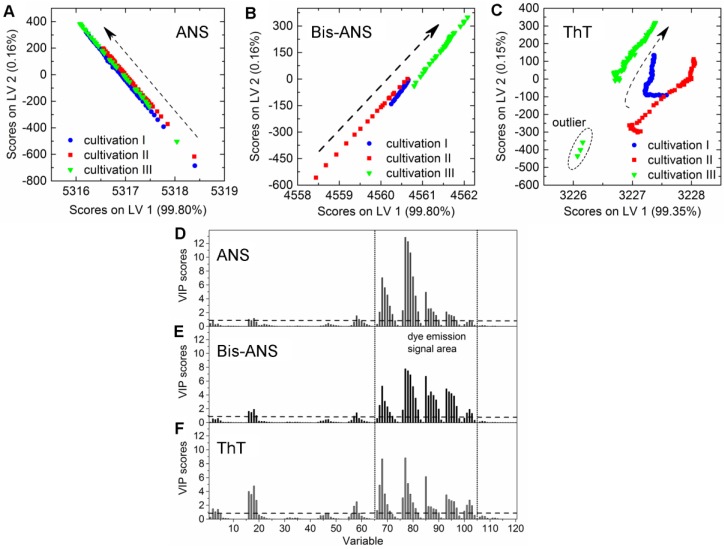
Scores plots of the PLS models for the prediction of aggregated mAb containing either (**A**) 100 µmol·L^−1^ ANS; (**B**) 2 µmol·L^−1^ Bis-ANS; or (**C**) 2 µmol·L^−1^ ThT. Dashed arrows indicate the development of the trajectories over cultivation time (only every 20th data point is shown). Furthermore, corresponding variable importance in projection (VIP) scores plots of the respective PLSR models are given in (**D**–**F**), respectively. LV: latent variable.

CHO cells expressing an aggregation-prone mAb were cultivated in 2-L bioreactors in fed-batch mode containing 100 µmol·L^−1^ ANS, 2 µmol·L^−1^ Bis-ANS, or 2 µmol·L^−1^ ThT, respectively, as medium additives. The cultivations were inoculated with high cell concentrations, and the extrinsic dyes were added after 67 ± 4 h in order to ensure antibody concentrations ≥20 mg·L^−1^. The selected dye concentrations were expected to be high enough to create additional recognizable fluorescence signals in the EEM. It was furthermore anticipated that these additional emission signals facilitate the calibration of PLSR models for the online prediction of mAb aggregate concentrations in CHO fed-batch cultivations. Culture conditions as well as dye and substrate concentrations in medium and feed are stated in Table 1. Samples were drawn from the bioreactor, and product concentrations and aggregate levels were determined following the method described by Paul et al. (2014) [25]. The PLSR models were calibrated with data recorded after dye addition. The final models were selected based on the quality parameters listed in Table 2 and their ability to predict the response variable based on the additionally recorded EEMs during cultivation. The trajectories on the scores plot of cultivations that contained either ANS or Bis-ANS showed a straight evolvement over the first two latent variables (LVs) (Figure 2A,B). Contrary to this, trajectories of the ThT cultivations indicated that besides the emission signals related to ThT, signals derived from CHO cells, medium, and feed also had an impact on the model (Figure 2C). This was demonstrated through the differences between the trajectories of the three fed-batch cultivations. It was not possible to increase the model quality through extensive preprocessing. Furthermore, outliers were identified in the scores plot of cultivation run III containing ThT. They were related to interfering light at the end of the cultivation (Figure 2C). 

**Figure 3:**
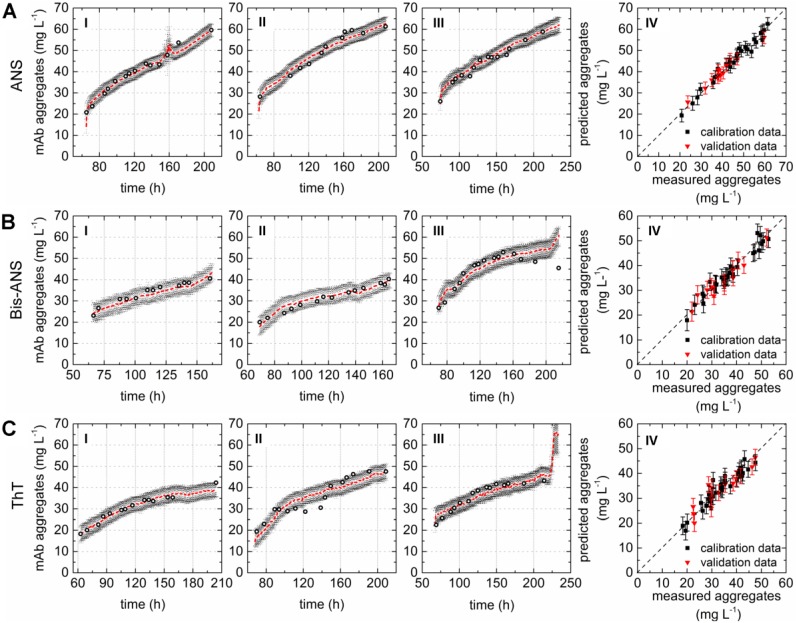
Correlation plots as a practical test by comparing offline values and values predicted based on the PLSR models calculated with the data recorded during three fed-batch cultivations (**I**–**III**) containing (**A**) 100 µmol·L^−1^ ANS; (**B**) 2 µmol·L^−1^ Bis-ANS; and (**C**) 2 µmol·L^−1^ ThT. Offline measured measurements (circles), predicted values (red), and corresponding confidence limits of the prediction based on the calculated errors (black). Plot IV (**A**–**C**) shows the predicted versus reference plots of the resulting PLSR models.

A direct comparison of the different PLS models can be done by comparing the respective variable importance in projection (VIP) scores plots. The importance of each wavelength pair for the projection in the model is shown by the VIP scores plots. Each recorded EEM can be vectorized into 120 wavelength pairs. Accordingly, the resulting PLSR model consisted of 120 variables. Variables with VIP scores close to 1 or higher are considered as important for the model [27,31]. The highest VIP scores in all three PLSR models were determined for the area between variable 66–105 (λ_ex_ 370–450 nm and λ_em_ 410–590 nm) of the VIP scores plot (Figure 2D–F). Furthermore, a second area between variable 16 and 28 (λ_ex_ 290 nm and λ_em_ 350–570 nm) showed VIP values close to one. More than 99% of the x- and y-variance were captured by 1 LVs of the PLSR models computed for cultivations containing ANS or Bis-ANS. However, R^2^_cal_ and R^2^_CV_ improved with the implementation of more LVs in both cases. Therefore, five LVs were selected for both models in order to meet the above proposed criteria of low RMSE’s, R^2^’s close to 1, together with the lowest possible number of LVs. Over 99% of the x-variance was already explained by LV1 of the ThT model, but only 95.2% of the variance in the y-dataset was captured. Furthermore, the R^2^_cal_ of this model improved with five additional LVs from <0.7 to 0.92. In general, the predicted and offline values of all cultivations and all models showed a close fit to the target line of the predicted versus measured plots (Figure 3). The PLSR model calculated for cultivations containing ANS showed the best correlations between offline values and predicted mAb aggregate concentrations (Figure 3A). An RMSEP of 1.7 mg·L^−1^ aggregated mAb was calculated for this model (Table 2). The Bis-ANS model performed in a similar way, but a higher RMSEP of 2.3 mg·L^−1^ mAb aggregates was calculated. Furthermore, an RMSEP of 3.1 mg·L^−1^ mAb aggregates was calculated for the ThT model. EEMs not included in the model calibration were used in a next step as input data for the aggregate prediction. Overall, a good correlation between measured and predicted values was observed (Figure 3), with some exception for Bis-ANS cultivation III and ThT cultivation III. For ThT cultivation III, the prediction assumed a rapid increase in product aggregation prior to harvest. For Bis-ANS cultivation III, the prediction indicated an increase in mAb aggregates, whereas the offline measured values suggested decreasing values.

## 4. Discussion

### 4.1. Fluorescence Dye Toxicity

Fluorescence dyes have so far been mainly used in protein purification and formulation development [32,33,34,35]. Their influence on cell growth and viability had to be investigated first in order to determine suitable concentrations of ANS, Bis-ANS, and ThT that can be added to CHO fed-batch cultures. Suitable dye concentrations were selected for further experiments based on the determined IC50s as well as on concentrations reported in literature. For example, Hawe et al. (2010) [35] studied the ability of 5 µmol·L^−1^ Bis-ANS to detect aggregation in commercially available therapeutic antibodies formulations. A suitable concentration of 50 µmol·L^−1^ ANS for the determination of antibody monomers and oligomers was proposed by Franey et al. (2010) [34]. Amani et al. (2014) used 10 µmol·L^−1^ ThT to investigate the influence of detergents addition on antibody conformation in formulations. Finally, Paul et al. (2015) used 10 µmol·L^−1^ Bis-ANS and 100 µmol·L^−1^ ThT in their at situ study. Their proposed high-throughput compatible method was able to distinguish between different amounts of mAb aggregates in cell culture supernatant. Based on literature and the toxicity experiments, it was expected that concentrations of 100 µmol·L^−1^ ANS, 2 µmol·L^−1^ Bis-ANS, and 2 µmol·L^−1^ ThT would be high enough to generate a distinct additional signal in the fluorescence scans. It became evident during the study that the selection of suitable dye concentrations is important, especially for high titer processes. The potential aggregate concentration and the used dye and its concentration have to be aligned, and might have to be determined for different production processes accordingly.

### 4.2. Extrinsic Dye-Related Emission Signals

The areas in the EEMs (λ_ex_ 370–450 nm and λ_em_ 410–590 nm) with increased fluorescence due to non-covalent dye aggregate interaction were identified based on the VIP scores plots (Figure 2D–F). The respective fluorescence areas were in accordance with Hawe et al. (2008b) [23], who proposed the same wavelengths for the applied dyes. The experiments also showed that fluorescence dye addition not only added a new emission signal to the EEMs, but also intensified the fluorescence signals of the whole scan. This became evident through the direct comparison of the EEM before and after the addition of the extrinsic dye to the cell culture. In general, the overall fluorescence signal intensity increased, and the area with the highest emission signals was dependent on the dye and its concentration (ANS > Bis-ANS > ThT). The resulting PLSR models were built based on so-called primary effects, since the non-covalent interaction of mAb aggregates and respective dye creates a fluorescence signal which is directly related to the y-dataset of the model. Interactions of the fluorescence dye and protein contained in the medium cannot be completely excluded. However, we already proved in previous work that PLSR models can be built for the prediction of mAb aggregates in cell culture supernatant using the extrinsic dyes Bis-ANS and ThT [16]. The selected PLSR models were able to predict the aggregate concentrations based on the recorded EEMs. Furthermore, predicted and measured concentrations were closely aligned in this work (Figure 3) and in the previously published work of Paul et al. (2015) [16]. Therefore, it can be concluded that an interaction of dye and media proteins was either compensated in the PLSR model or it was rather negligible.

### 4.3. Chemometric Modeling

Fluorescence signals derived from non-covalent interactions between hydrophobic regions of the mAb aggregates and either ANS or Bis-ANS created high emission signal intensities over a broad range in the EEMs. These signals were successfully correlated via PLSR to the aggregated mAb concentration. The fluorescent signals created by these dyes superimposed the fluorescence of feed medium and cell culture in specific areas of the EEM, as discussed earlier. This implied that the fluorescent feed medium had no impact on the fluorescence signals relevant for mAb aggregates. Furthermore, the score values suggested that the fluorescence increase of certain wavelength pairs was directly related to the increasing aggregate concentration. As a result, the calibration of PLSR models for the prediction of aggregated mAb based on extrinsic signals was feasible. In contrast, ThT was identified to be more toxic than ANS or Bis-ANS. Therefore ThT was only used in a rather low concentration compared to Paul et al. (2015) [16], which resulted in low fluorescence signal intensities in the area of λ_ex_ 370–450 nm and λ_em_ 410–590 nm. In contrast, the same Bis-ANS concentration generated higher fluorescence intensities in the same EEM area. However, the dye-related fluorescence signals did not completely outshine the signals generated through the fluorescent feed medium and cell culture. Therefore, a second region in the VIP scores plot showed its relevance for the model generation. The area of λ_ex_ 290 nm and λ_em_ 350–570 nm showed VIP values close to one, which can be related to the intrinsic fluorescence of the total protein concentration (Figure 2). This supported the assumption that the chemometric model was based on a combination of extrinsic signals which can also be called primary effects related to the fluorescence signal of ThT and intrinsic signals, so-called secondary effects of the culture and medium. We furthermore proved in earlier work that the addition of fluorescent feed did not hinder model generation [27]. The scores plots and the predictions identified some EEM of Bis-ANS cultivation run III and ThT cultivation run III as possible outliers (Figure 3). One reason could be a biased prediction due to interfering light because of scattering effects [27,36]. Another explanation could be mAb aggregate precipitation during sample preparation. This was already seen by Paul et al. (2014) [25], who described that large aggregates can be formed. These higher molecular weight species might be removed from the sample through centrifugation or filtration. From this study, it can be concluded that ANS was evaluated as the most promising candidate for this application, since the PLSR model showed high predictive power. Furthermore, the result indicated that the dye concentration of 100 mmol·L^−1^ could even be reduced. However, it is not applicable to use extrinsic dyes as media components in commercial biopharmaceutical production processes, since this would trigger questions concerning the product safety. Nevertheless, in some cases it could be beneficial to use fluorescence dyes to generate dominant signals for the chemometric modeling of mAb aggregate concentrations. It could be used in scaled-down models during media and process development or during clone selection screenings. It could be furthermore beneficial for early process development stages or during troubleshooting in order to identify raw materials that are suspected to trigger protein aggregation. The use of extrinsic dyes for late-stage process development is not feasible. Therefore, whether the intrinsic fluorescence of cell culture, medium, and product can be used to set up soft sensors for the prediction of antibody aggregation must be evaluated in further work.

## Figures and Tables

**Table 1:** Culture conditions for all fed-batch fermentations expressing a full-size monoclonal antibody in the presence of extrinsic dyes. ANS: 1-anilinonaphthalene-8-sulfonate; Bis-ANS: 4,4′-bis-1-anilinonaphthalene-8-sulfonate; mAb: monoclonal antibody; Th T: thioflavin T.

	Start Concentration						
Cultivation	Glucose (g·L^−1^)	Glutamine (mmol·L^−1^)	Cultivation Time (h)	Time of Dye Addition (h)	X_V_ max (×10^6^ mL^−1^)	mAb (mg·L^−1^)	Aggregated mAb (mg·L^−1^)
ANS I	1.63	1.26	208	64	5.06	95	60
ANS II	1.90	1.75	208	63	4.33	89	61
ANS III	2.84	2.03	212	74	3.38	88	59
Bis-ANS I	1.92	2.14	161	66	4.86	61	41
Bis-ANS II	2.37	1.93	165	69	4.55	57	40
Bis-ANS III	2.40	2.02	215	70	3.00	71	45
Th T I	1.99	2.08	204	63	4.86	58	42
Th T II	1.93	1.91	209	68	4.06	65	48
Th T III	2.84	2.03	214	67	3.67	59	43

**Table 2:** Partial least square regression (PLSR) modeling results for the prediction of aggregated mAb concentrations. EPO: External parameter orthogonalization; MSC: multiplicative signal correction.

			Calibration		Cross-Validation		Prediction	
Extrinsic Dye	Preprocessing	LV	R^2^_cal_	RMSEC [mg·mL^−1^]	R^2^_CV_	RMSECV [mg·mL^−1^]	R^2^_P_	RMSEP [mg·mL^−1^]
ANS	MSC (mean) EPO (1 PC)	5	0.97	1.1	0.94	2.6	0.97	1.7
Bis-ANS	MSC (mean) EPO (1 PC)	5	0.96	1.9	0.89	3.2	0.92	2.3
ThT	MSC (mean)	6	0.92	2.2	0.85	3.0	0.85	3.1
